# Epidemiology and antimicrobial susceptibility of *Staphylococcus aureus* in children in a tertiary care pediatric hospital in Milan, Italy, 2017—2021

**DOI:** 10.1186/s13052-022-01262-1

**Published:** 2022-05-07

**Authors:** Adriano La Vecchia, Giulio Ippolito, Vittoria Taccani, Elisabetta Gatti, Patrizia Bono, Silvia Bettocchi, Raffaella Pinzani, Claudia Tagliabue, Samantha Bosis, Paola Marchisio, Carlo Agostoni

**Affiliations:** 1 University of Milan, Milan, Italy; 2 Laboratory of Microbiology, Fondazione IRCCS Ca’ Granda Ospedale Maggiore Policlinico, Milan, Italy; 3 Pediatric Emergency Department, Fondazione IRCCS Ca’ Granda Ospedale Maggiore Policlinico, Milan, Italy; 4 De Marchi Foundation, Pediatric Area, Fondazione IRCCS Ca’ Granda Ospedale Maggiore Policlinico, Milan, Italy; 5 Fondazione IRCCS Ca’ Granda Ospedale Maggiore Policlinico, Pediatric Highly Intensive Care Unit, Milan, Italy; 6 Department of Pathophysiology and Transplantation, University of Milan, Milan, Italy; 7 Fondazione IRCCS Ca’ Granda Ospedale Maggiore Policlinico, Pediatric Intermediate Care Unit, via Francesco Sforza 9, 20122 Milan, Italy; 8 Department of Clinical Sciences and Community Health (DISCCO), University of Milan, Milan, Italy

**Keywords:** Staphylococcus aureus, MRSA, MSSA, Antimicrobial susceptibility, Resistance, Children, Public health, Surveillance, COVID-19 pandemic

## Abstract

**Background:**

Methicillin-resistant *Staphylococcus aureus* (MRSA) is highly prevalent worldwide and can cause severe diseases. MRSA is associated with other antibiotic resistance. COVID-19 pandemic increased antimicrobial resistance in adult patients. Only a few data report the antimicrobial susceptibility of *S. aureus* in the Italian pediatric population, before and during the COVID-19 pandemic.

**Methods:**

We included all the *S. aureus* positive samples with an available antibiogram isolated from pediatric patients (< 18 years old) in a tertiary care hospital in Milan, Italy, from January 2017 to December 2021. We collected data on demographics, antimicrobial susceptibility, and clinical history. We compared methicillin-susceptible *Staphylococcus aureus* (MSSA) and MRSA strains. We calculated the frequency of isolation by year. The incidence of isolates during 2020 was compared with the average year isolation frequency using the univariate Poisson test. We compared the proportion of MRSA isolates during 2020 to the average proportion of other years with the Chi-squared test.

**Results:**

Our dataset included a total of 255 *S. aureus* isolated from 226 patients, 120 (53%) males, and 106 (47%) females, with a median age of 3.4 years (IQR 0.8 – 10.5). The mean isolation frequency per year was 51. We observed a significant decrease of isolations during 2020 (*p* = 0.02), but after adjusting for the total number of hospitalization per year there was no evidence that the incidence changed. Seventy-six (30%) *S. aureus* were MRSA. Twenty (26%) MRSA vs 23 (13%) MSSA (*p* = 0.02) were hospital-acquired. MRSA strains showed higher resistance to cotrimoxazole, clindamycin, macrolides, levofloxacin, gentamicin, and tetracyclin than MSSA strains. None of MRSA were resistant to linezolid and vancomycin, one was resistant to daptomycin. The proportion of MRSA did not change during the COVID-19 pandemic. The overall clindamycin resistance was high (17%). Recent antibiotic therapy was related to MRSA infection.

**Conclusion:**

The proportion of MRSA did not change during the COVID-19 pandemic and remained high. Clindamycin should not be used as an empirical MRSA treatment due to its high resistance.

## Background

*Staphylococcus aureus* is a major source of disease ranging from soft-tissue infections (SSTIs) to bacteremia, associated with substantial morbidity and mortality in hospital settings and communities and also affecting children [1, 2].

Methicillin-resistant *S.aureus* (MRSA) strains are highly prevalent, and have traditionally been confined to individuals with frailty such as recent hospitalization or health-care utilization, residence in long-term care units, hemodialysis, indwelling percutaneous medical device and catheters [3, 4].

However, cases of infections caused by MRSA have also been documented among healthy community members, particularly among children without established risk factors for MRSA acquisition. These infections are mostly acquired in the community and have been referred to as community-associated MRSA infections (CA-MRSA) [5, 6].

CA-MRSA are mainly responsible for skin and soft tissue infections such as cellulitis or abscess. Health care-associated MRSA (HCA-MRSA) and hospital-acquired MRSA (HA-MRSA) share similar characteristics [4] and predominantly cause pneumonia, sepsis, or serious conditions [7, 8].

The prevalence of MRSA strains has not been adequately quantified in many countries, including Italy, especially in children [9, 10]. Limited data compared different patterns of antibiotic resistance complicated by the limited number of effective antibiotics analyzed in children [11]. In addition, recurrent infections, common in children, can change antibiotic sensitivity [12].

The guidelines highlight the necessity of using different antibiotic empiric therapies depending on the local prevalence of resistance pattern [13, 14], but the general inadequate knowledge makes complex the management of MRSA.

Knowledge of local antibiotic resistance patterns represents a major challenge in the management of MRSA infection, also considering that antibiotic control in children may affect antibiotic resistance in adults [15, 16].

The COVID-19 pandemic increased antimicrobial resistance in adult patients, mainly due to inappropriate antibiotic prescription [17]. A single-centre study in a pediatric tertiary care hospital in London, United Kingdom, found an increase in crude antibiotic consumption during the COVID-19 pandemic, but the association disappeared after adjusting for case-mix [18]. Moreover, the extensive use of telehealth [19], social distancing, and school closure could have led to a decrease in the transmission of bacterial infections [20] with the consequent low antibiotic prescription rate in primary care.

The present investigation considers the demographic and clinical features of patients with MRSA and the corresponding antimicrobial pattern before and during the COVID-19 pandemic in a pediatric tertiary hospital, which functioned as a hub for SARS-CoV-2 positive patients.

## Methods

### Data source and study design

We performed an observational study at a tertiary care hospital (IRCCS Fondazione Cà Granda Ospedale Maggiore Policlinico), in Milan, northern Italy, from January 2017 to December 2021.

We included all the *S. aureus* positive samples for which an antibiogram was performed gathered in the microbiology electronic record, isolated from pediatric patients (< 18 years old). We collected data on age, sex, antimicrobial susceptibility, medical history, clinical course, and hospitalization days. *S. aureus* isolated after 48 h of hospitalization were considered hospital-acquired, those isolated from patients receiving hemodialysis, chemotherapy, and recent (within 30 days) surgery or exposure to health care were considered health care-associated, the others community-acquired.

The Milano Area 2 Ethical Committee approved the study, which included a waiver of informed consent because of the retrospective nature of the investigation.

### Laboratory test

The samples were collected from blood cultures, skin, nose, oropharyngeal, ear, conjunctival, preputial, wound, exudate, and rectal swabs, lymph node, fistula fluid, synovial fluid, peritoneal fluid, bone samples, and urine samples gathered from the stoma, urine bag or sterile collection cups.

Traditional culture and susceptibility testing took between 48 and 72 h, including a 16 to 24 h incubation and another 16 to 24 h to complete the susceptibility tests. Pathogen identification was confirmed by MALDI-TOF MS technology performed using VITEK® MS Plus (Biomerieux SA).

Antimicrobial susceptibility for antibiotics including cefoxitin, clindamycin, cotrimoxazole, linezolid, tetracycline, macrolides, daptomycin, levofloxacin, fusidic acid, mupirocin, gentamycin, and vancomycin was assessed using a specific card for Gram-positive bacteria analyzed with automated instrument Vitek 2 (Biomerieux SA). Cefoxitin (cefoxitin screening) is a very sensitive and specific marker of mecA/mecC-mediated methicillin resistance and is the antibiotic of choice to define the MRSA strain.

The Gram staining technique was only performed for positive blood cultures, to discriminate between Gram + and – in a short period to start a targeted therapy as soon as possible.

When blood culture was positive, in clinically suspected cases requested by the pediatrician, we used the molecular methods IDI-MRSA kit on a Smart Cycler II thermal cycler instrument (Cepheid, Sunnyvale, Calif., USA) or Xpert® MRSA/SA Blood Culture on GeneXpert instrument (Cepheid, Sunnyvale, Calif., USA) to distinguish MRSA strains from methicillin-susceptible *Staphylococcus aureus* (MSSA).

### Statistical analysis

Continuous data are presented as median and interquartile range. We compared MSSA and MRSA strains. The chi-square test or Fisher’s exact test were used for categorical variables, the Mann–Whitney U-test for continuous ones. The incidence of isolations during 2020 was compared with the average year isolation frequency using the univariate Poisson test. We compared the proportion of MRSA isolations during 2020 with the average proportion of other years with the Chi-squared test. Statistical significance was considered as a *p*-value < 0.05. Statistical analysis was performed using R software (version 3.6.3 for Windows).

## Results

Our dataset included a total of 255 *S. aureus* isolated from 226 patients, 120 (53%) male, and 106 (47%) female, with a median age at the isolation of 3.4 years (IQR 0.8 – 10.5). Overall, 147 (58%) *S. aureus* were cultured from SSTIs, 48 (19%) from upper respiratory tract infections (URTIs), 35 (14%) from bloodstream infections, 10 (4%) from acute conjunctivitis, 6 (2%) from urinary tract infections (UTIs), 5 (2%) from osteoarticular infections and 4 (2%) from infections in other sterile sites. Forty-three (17%) samples were obtained after two days of hospitalization, 59 (23%) were health care-associated infections, while the remaining 153 (60%) were considered community-acquired. The mean isolation frequency per year was 51. We isolated 41 (16%) *S. aureus* in 2017, 65 (25%) in 2018, 50 (20%) in 2019, 36 (14%) in 2020 and 63 (25%) in 2021. We observed a significant decrease in absolute isolation during 2020 (*p* = 0.02). However, when the number of isolations per year was adjusted for the total number of hospitalizations during the same year, 2228 during 2020 versus an average hospitalization per year of 2994, we found a proportion of 16:1000 during 2020 versus an average of 17:1000.

We identified a predisposing factor in 92 (36%) cases. The most prevalent factor in our cohort was central venous catheter (*n* = 22), followed by trauma (*n* = 15), peripheral venous catheter (*n* = 9) and surgical intervention (*n* = 9). In 95 (34%) cases there was at least a chronic disease. The most prevalent was epidermolysis bullosa (*n* = 23), followed by neurologic diseases (*n* = 15), atopic dermatitis (*n* = 13), kidney diseases (*n* = 13) and metabolic diseases (*n* = 6). Patients with chronic illness had significantly higher proportions of health care-associated infections (56%) compared to other patients (4%, *p* < 0.01). There was no significant difference in the proportions of hospital-acquired infections (19% vs 16%).

Fifty-eight (23%) cases had a coinfection. Sixty-nine (27%) cases had a positive anamnesis for recent antibiotic use, 2 (1%) for antibiotic prophylaxis. The most frequently used antibiotics were amoxicillin and clavulanic acid (*n* = 40) followed by ampicillin (*n* = 5), ceftriaxone (*n* = 5) and azithromycin (*n* = 4). The median value of the maximal C reactive protein (CRP) measured during hospitalization was 1.85 mg/dl (IQR 0.3 – 5.8). The median length of hospital stay was 11 days (IQR 7 – 19). Comparing the patients with positive versus negative anamnesis for recent antibiotic use, there were no differences between the median age (3.3 and 3.3 years), maximal CRP in mg/dl (2 and 1.5 respectively), and days of hospitalization (14 and 10 respectively). Eighteen (45%) of the 40 cases with a positive anamnesis for recent amoxicillin-clavulanic acid use had an MRSA infection, the proportion being significantly higher than the other cases (*p* = 0.02).

Seventy-six (30%) *S. aureus* were MRSA. Table 1 summarizes the antimicrobial susceptibility pattern.

**Table 1:** Antimicrobial susceptibility pattern. Data are presented as numbers (percentages)

	***Susceptible***	***Resistant***	***Intermediate***	***Not tested***
Methicillin	179 (70)	76 (30)	0 (0)	0 (0)
Cotrimoxazole	222 (87)	28 (11)	2 (1)	3 (1)
Clindamycin	202 (79)	43 (17)	7 (3)	3 (1)
Macrolides	190 (75)	59 (23)	0 (0)	6 (2)
Linezolid	250 (98)	0 (0)	0 (0)	5 (2)
Daptomycin	247 (97)	1 (0.4)	0 (0)	7 (3)
Levofloxacin	203 (79)	46 (18)	0 (0)	6 (2)
Fusidic Acid	209 (82)	37 (14)	0 (0)	9 (4)
Mupirocin	233 (91)	14 (5)	0 (0)	8 (3)
Gentamicin	214 (84)	35 (14)	0 (0)	6 (2)
Tetracycline	223 (87)	23 (9)	4 (2)	5 (2)
Vancomycin	255 (100)	0 (0)	0 (0)	0 (0)

Twenty (26%) MRSA versus 23 (13%) MSSA (*p* = 0.02) were hospital-acquired, 17 (22%) MRSA versus 42 (23%) MSSA were health care-associated (*p* = 0.9). Compared to MSSA, MRSA infections presented significant higher proportions of positive anamnesis for recent antibiotic use (40% vs 25%, *p* = 0.01) and higher median value of maximal CRP (3.6 mg/dl, IQR 0.9 – 5.7, vs 1.1, IQR 0.2 – 5.9, *p* = 0.045). We did not observe other significant differences in demographic, clinical characteristics and site of infection between MRSA and MSSA Table 2.

**Table 2:** Demographic**,** clinical characteristics, and site of infections of patients according to methicillin resistance

	***MRSA (n* = *76)***	***MSSA (n* = *179)***	***P-value***
**Demographics**			
Male	34 (45)	94 (53)	0.2
Female	42 (55)	84 (47)	0.2
Age (Years)	3.5 [0.7–7.7]	3.3 [0.9–11.2]	0.4
**Clinical characteristics**			
Predisposing factor	25 (33)	67 (37)	0.5
Chronic disease	29 (38)	66 (37)	0.8
Recent antibiotic use	29 (40)	42 (25)	0.01
Hospital-acquired	20 (26)	23 (13)	0.02
Health care-associated	17 (22)	42 (23)	0.8
Maximal CRP (mg/dl)	3.6 [0.9–5.7]	1.1 [0.2–5.9]	0.045
Hospitalization days	15 [8.5–21.5]	10 [6–16.2]	0.1
**Site of infections**			
SSTIs	43 (57)	104 (58)	0.7
Bloodstream infections	13 (17)	22 (12)	0.3
URTIs	12 (16)	36 (20)	0.4
Acute conjunctivitis	3 (4)	7 (4)	1
UTIs	4 (5)	2 (1)	0.07
OAIs	0 (0)	5 (3)	0.3
Others sites	1 (1)	3 (2)	1

Data are presented as numbers (percentages) or median [interquartile range]

*MRSA* Methicillin-resistant *Staphylococcus aureus, MSSA* Methicillin-susceptible *Staphylococcus aureus, OAIs* Osteoarticular infections, Sstis Soft tissue infections, *URTI* Upper respiratory tract infections, *UTIs* Urinary tract infections

There was higher resistance of MRSA versus MSSA to cotrimoxazole (22% vs 6%), clindamycin (24% vs 14%), macrolides (37% vs 17%), levofloxacin (46% vs 6%), fusidic acid (34% vs 6%), gentamicin (33% vs 6%), and tetracyclin (20% vs 4%). None of MRSA were resistant to linezolid and vancomycin, one was resistant to daptomycin. Table 3 shows the antimicrobial resistance pattern of MRSA and MSSA.

**Table 3:** Antimicrobial susceptibility pattern according to methicillin resistance

		***Cotrimoxazole***	***Clindamycin***	***Macrolides***	***Linezolid***	***Daptomycin***	***Levofloxacin***	***Fusidic Acid***	***Mupirocin***	***Gentamicin***	***Tetracyclin***	***Vancomycin***
**MRSA**	**S**	56 (74)	53 (70)	46 (60)	75 (99)	72 (95)	40 (53)	47 (62)	68 (89)	50 (66)	57 (75)	76 (100)
	**R**	17 (22)	18 (24)	28 (37)	0 (0)	1 (1)	35 (46)	26 (34)	6 (8)	25 (33)	15 (20)	0 (0)
	**I**	2 (3)	4 (5)	0 (0)	0 (0)	0 (0)	0 (0)	0 (0)	0 (0)	0 (0)	4 (5)	0 (0)
	**NT**	1 (1)	1 (1)	2 (3)	1 (1)	3 (4)	1 (1)	3 (4)	2 (3)	1 (1)	0 (0)	0 (0)
**MSSA**	**S**	166 (93)	149 (83)	144 (80)	175 (98)	175 (98)	163 (91)	162 (90)	165 (92)	164 (92)	166 (93)	176 (100)
	**R**	11 (6)	25 (14)	31 (17)	0 (0)	0 (0)	11 (6)	11 (6)	8 (4)	10 (6)	8 (4)	0 (0)
	**I**	0 (0)	3 (2)	0 (0)	0 (0)	0 (0)	0 (0)	0 (0)	0 (0)	0 (0)	0 (0)	0 (0)
	**NT**	2 (1)	2 (1)	4 (2)	4 (2)	4 (2)	5 (3)	6 (3)	6 (3)	5 (3)	5 (3)	0 (0)
***P*-value**		< 0.01	0.03	< 0.01	1	0.3	< 0.01	< 0.01	0.4	< 0.01	< 0.01	1

Data are presented as numbers (percentages)

*MRSA* Methicillin-resistant Staphylococcus aureus, *MSSA* Methicillin-susceptible Staphylococcus aureus, *S* Sensible, *R* Resistant, *I* Intermediate, *NT* Not tested

The proportion of MRSA by year was highest (36.5%) in 2017 and lowest (19.4%) in 2020, with intermediate values of 30.8%, 30.2% and 30% in 2018, 2021 and 2019 respectively. The difference was not significant (*p* = 0.1). The 2020 cases did not have significant difference in the proportion of chronic conditions (42% vs 36%, *p* = 0.6) compared with the others 4 years cases, and in the site of infection with 55% vs 58% SSTIs (*p* = 0.8), 14% vs 14% bloodstream infections (*p* = 1), 11% vs 20% URTIs (*p* = 0.2), 3% vs 2% osteoarticular infections (*p* = 0.5), 6% vs 2% UTIs (*p* = 0.2) and 6% vs 4% acute conjunctivitis (*p* = 0.6).

## Discussion

To our knowledge, this is the first Italian study to determine the *S. aureus* antimicrobial susceptibility and the prevalence of MRSA in a pediatric hospital setting. We found a high proportion of MRSA (30%). The prevalence of MRSA is similar to that reported in an Italian study on the adult population (33.6%) [21] and to the one recently estimated by the Antimicrobial Resistance Collaborators in Italy in 2019 (between 30 and 40%) [22], but higher than the one reported in a Turkish pediatric study (18.2%) [23]. The stable trend in the proportion of MRSA in our pediatric sample is consistent with the stable/increasing trend in the Italian general population (from 34.1% in 2015 to 35.6% in 2019) reported by the European Centre for Disease Prevention and Control (ECDC) in the Annual Epidemiological Report for 2019 [24].

MRSA strains showed a significantly increased resistance to cotrimoxazole, clindamycin, macrolides, levofloxacin, gentamicin, and tetracyclin than MSSA strains. These results are consistent with data in the adult population, which reported that MRSA strains were more resistant to all antibiotics except for doxycycline [25] and significantly more resistant to ciprofloxacin, clindamycin, erythromycin, rifampicin, and tetracycline [21]. We reported a high overall prevalence of clindamycin resistance (17%). Accordingly, clindamycin should not be used for empirical MRSA treatment, in line with the 10% limit indicated in the Clinical Practice Guidelines by the Infectious Diseases Society of America [14]. As far as the intravenous route, we did not find *S. aureus* resistant to vancomycin and linezolid, and only one MRSA strain resistant to daptomycin. Therefore, these antibiotics should be used in severe infections as indicated in the above-quoted guidelines [14]. We reported a low prevalence of mupirocin resistance (5%), consistent with a Brazilian study that found a 1.1% resistance [26]. In contrast with the aforementioned study that reported a low prevalence of fusidic acid resistance (5.9%) [26], we found a higher proportion of resistant strains (14%). Topical mupirocin can thus be used for children with minor skin infections and secondarily infected skin lesions [14].

The World Health Organization (WHO) indicates surveillance as the core pillar in the global action plan on microbial resistance [27]. A critical factor associated with MRSA is the quality of prescribed antibiotics, especially considering their spectrum of activity [28]. Antibiotics are the most frequently prescribed drug in children [29] with a high rate of inappropriate prescription [30]. Efforts should focus on decreasing broad-spectrum antibiotic use for common diseases [31]. Italy has one of the highest rates of antibiotic use in the pediatric population among high-income countries, and the prescriptions pattern is dominated by broad-spectrum agents [32]. Despite the diffusion of national clinical guidelines, inappropriate antibiotic prescriptions for common diseases such as acute otitis media and bronchiolitis remain frequent [33, 34]. The MAREA study monitored the adherence to guidelines of antibiotic use in pediatric pneumonia in Liguria, North-West Italy, finding a global adherence to guidelines in 45% of cases and 61% of patients receiving multidrug empirical regimens. The adherence to guidelines improved after a 1 day-educational intervention on pediatricians [35]. We reported a relation between a positive anamnesis for recent antibiotic use and MRSA infections, and the relation was even stronger when we considered amoxicillin-clavulanic acid use. In our sample, the most common antibiotics were broad spectrum such as amoxicillin-clavulanate, ampicillin, ceftriaxone, and azithromycin. Anyway, our data do not allow us to assess the appropriateness of prescriptions.

The total number of *S. aureus* isolations significantly decreased during 2020, but after adjusting for the total number of hospitalization per year there was no evidence that the incidence changed. The drop in hospitalizations is due to the increased use of telehealth [19] and the substantial decrease in pediatric hospitalizations for many acute conditions, such as bronchiolitis and asthma, during the first and second wave of the COVID-19 pandemic [36, 37]. The role of predisposition in colonized patients, environmental factors, and antibiotic pressure in S. aureus hospital and household transmission is not fully understood [38], although the household environment plays a key role in MRSA transmission [39]. Next-generation sequencing and whole genomic sequencing are promising methods for the control of nosocomial infections [38, 40].

Since we performed a retrospective analysis focusing on strains originating from a single-center, it was not possible to differentiate infections from colonization. Moreover, we had a high prevalence of epidermolysis bullosa patients, since our facility is a referral center for this rare genodermatosis. A strength of our work is a relatively large dataset and the uniform and comparative analytic approach.

## Conclusion

Surveillance of antimicrobial resistance is essential to improve infection control, antibiotic prescriptions, and preventions policies [21, 27]. Our results did not show changes in MRSA proportions during the COVID-19 pandemic. The proportion of MRSA remains high. In our context, clindamycin should not be used as an empirical MRSA treatment due to its high resistance [14]. Recent antibiotic use was associated with higher proportions of MRSA infections. The efforts should focus on decreasing broad-spectrum antibiotic use for common diseases.

## Data Availability

Data are available at the corresponding author upon reasonable request.

## Abbreviations

CA: Community-acquired
COVID-19: Coronavirus disease 19
CRP: C reactive protein
ECDC: European Centre for Disease Prevention and Control
HA: Hospital-acquired
HCA: Health care-associated
IQR: Interquartile range
MRSA: Methicillin-resistant *Staphylococcus aureus*
MSSA: Methicillin-susceptible *Staphylococcus aureus*
OAIs: Osteoarticular infections
SSTIs: Soft tissue infections
URTI: Upper respiratory tract infections
UTIs: Urinary tract infections
WHO: World Health Organization

## References

[1] Iwatsuki K, Yamasaki O, Morizane S, Oono T (2006). Staphylococcal cutaneous infections: Invasion, evasion and aggression. J Dermatol Sci.

[2] Turner NA, Sharma-Kuinkel BK, Maskarinec SA, Eichenberger EM, Shah PP, Carugati M (2019). Methicillin-resistant Staphylococcus aureus: an overview of basic and clinical research. Nat Rev Microbiol.

[3] Fernando SA, Gray TJ, Gottlieb T (2017). Healthcare-acquired infections: prevention strategies. Intern Med J.

[4] Lesens O, Hansmann Y, Brannigan E, Hopkins S, Meyer P, O'Connel B (2005). Healthcare-associated staphylococcus aureus bacteremia and the risk for methicillin resistance: is the centers for disease control and prevention definition for community-acquired bacteremia still appropriate?. Infect Control Hosp Epidemiol.

[5] Mediavilla JR, Chen L, Mathema B, Kreiswirth BN (2012). Global epidemiology of community-associated methicillin resistant Staphylococcus aureus (CA-MRSA). Curr Opin Microbiol.

[6] Otto M (2013). Community-associated MRSA: What makes them special?. Int J Med Microbiol.

[7] Boucher HW, Corey GR (2008). Epidemiology of methicillin-resistant staphylococcus aureus. Clin Infect Dis.

[8] Naimi TS (2003). Comparison of community- and health care-associated methicillin-resistant staphylococcus aureus infection. JAMA.

[9] David MZ, Daum RS, Bayer AS, Chambers HF, Fowler VG, Miller LG (2014). Staphylococcus aureus bacteremia at 5 US academic medical centers, 2008–2011: significant geographic variation in community-onset infections. Clin Infect Dis.

[10] Grundmann H, Aanensen DM, van den Wijngaard CC, Spratt BG, Harmsen D, Friedrich AW (2010). Geographic distribution of staphylococcus aureus causing invasive infections in Europe: a molecular-epidemiological analysis. PLoS Med.

[11] Wattal C, Goel N (2019). Pediatric blood cultures and antibiotic resistance: an overview. Indian J Pediatr.

[12] Medernach RL, Logan LK (2018). The growing threat of antibiotic resistance in children. Infect Dis Clin North Am.

[13] Esposito S, Bassetti M, Concia E, De Simone G, De Rosa FG, Grossi P, Diagnosis and management of skin and soft-tissue infections (SSTI) (2017). A literature review and consensus statement: an update. J Chemother.

[14] Liu C, Bayer A, Cosgrove SE, Daum RS, Fridkin SK, Gorwitz RJ (2011). Clinical practice guidelines by the infectious diseases society of America for the treatment of methicillin-resistant staphylococcus aureus infections in adults and children: executive summary. Clin Infect Dis.

[15] Bielicki JA, Lundin R, Sharland M (2015). Antibiotic resistance prevalence in routine bloodstream isolates from children’s hospitals varies substantially from adult surveillance data in Europe. Pediatr Infect Dis J.

[16] Cassini A, Högberg LD, Plachouras D, Quattrocchi A, Hoxha A, Simonsen GS (2019). Attributable deaths and disability-adjusted life-years caused by infections with antibiotic-resistant bacteria in the EU and the European Economic Area in 2015: a population-level modelling analysis. Lancet Infect Dis.

[17] Lai C, Chen S, Ko W, Hsueh P (2021). Increased antimicrobial resistance during the COVID-19 pandemic. Int J Antimicrob Agents.

[18] Vestesson E, Booth J, Hatcher J, McGarrity O, Sebire N, Steventon A (2022). The impact of the COVID-19 pandemic on antimicrobial prescribing at a specialist paediatric hospital: an observational study. J Antimicrob Chemother.

[19] Curfman A, McSwain S, Chuo J, Yeager-McSwain B, Schinasi D, Marcin J (2021). Pediatric Telehealth in the COVID-19 Pandemic Era and Beyond. Pediatrics.

[20] McNeil J, Flores A, Kaplan S, Hulten K (2021). The indirect impact of the SARS-CoV-2 pandemic on invasive group a streptococcus, streptococcus pneumoniae and staphylococcus aureus infections in Houston area children. Pediatr Infect Dis J.

[21] Pignataro D, Foglia F, Della Rocca MT, Melardo C, Santella B, Folliero V (2020). Methicillin-resistant staphylococcus aureus: epidemiology and antimicrobial susceptibility experiences from the university hospital ‘Luigi Vanvitelli’ of Naples. Path Glob Health.

[22] Murray C, Ikuta K, Sharara F, Swetschinski L, Robles Aguilar G, Gray A (2022). Global burden of bacterial antimicrobial resistance in 2019: a systematic analysis. The Lancet.

[23] Arikan K, Karadag-Oncel E, Aycan AE, Yuksekkaya S, Sancak B, Ceyhan M (2020). Epidemiologic and molecular characteristics of staphylococcus aureus strains isolated from hospitalized pediatric patients. Pediatr Infect Dis J.

[24] Antimicrobial resistance in the EU/EEA (EARS-Net) - Annual Epidemiological Report for 2019. European Centre for Disease Prevention and Control. 2021 [cited 29 Dec 2021]. Available from: https://www.ecdc.europa.eu/en/publications-data/surveillance-antimicrobial-resistance-europe-2019

[25] Horváth A, Dobay O, Sahin-Tóth J, Juhász E, Pongrácz J, Iván M (2020). Characterisation of antibiotic resistance, virulence, clonality and mortality in MRSA and MSSA bloodstream infections at a tertiary-level hospital in Hungary: a 6-year retrospective study. Ann Clin Microbiol Antimicrob.

[26] Bessa GR, Quinto VP, Machado DC, Lipnharski C, Weber MB, Bonamigo RR (2016). Staphylococcus aureus resistance to topical antimicrobials in atopic dermatitis. An Bras Dermatol.

[27] “Surveillance.” www.euro.who.int, www.euro.who.int/en/health-topics/disease-prevention/antimicrobial-resistance/surveillance. (Accessed in 27 Dec 2021).

[28] Borg MA, Camilleri L (2021). What is driving the epidemiology of methicillin-resistant staphylococcus aureus infections in Europe?. Microb Drug Resist.

[29] Sturkenboom MC, Verhamme KM, Nicolosi A, Murray ML, Neubert A, Caudri D (2008). Drug use in children: cohort study in three European countries. BMJ.

[30] Barry E, Smith SM (2015). Potentially inappropriate prescribing in children. Fam Pract.

[31] Vaz LE, Kleinman KP, Raebel MA, Nordin JD, Lakoma MD, Dutta-Linn MM (2014). Recent trends in outpatient antibiotic use in children. Pediatrics.

[32] de Bie S, Kaguelidou F, Verhamme KM, De Ridder M, Picelli G, Straus SM (2016). Using prescription patterns in primary care to derive new quality indicators for childhood community antibiotic prescribing. Pediatr Infect Dis J.

[33] Palma S, Rosafio C, Del Giovane C, Patianna V, Lucaccioni L, Genovese E (2015). The impact of the Italian guidelines on antibiotic prescription practices for acute otitis media in a paediatric emergency setting. Ital J Pediatr.

[34] Biagi C, Scarpini S, Paleari C, Fabi M, Dondi A, Gabrielli L (2021). Impact of guidelines publication on acute bronchiolitis management: 10-year experience from a tertiary care center in Italy. Microorganisms.

[35] Di Pietro P, Della Casa Alberighi O, Silvestri M, Tosca M, Ruocco A, Conforti G (2017). Monitoring adherence to guidelines of antibiotic use in pediatric pneumonia: the MAREA study. Ital J Pediatr.

[36] Wilder J, Parsons C, Growdon A, Toomey S, Mansbach J (2020). Pediatric hospitalizations during the COVID-19 pandemic. Pediatrics.

[37] Ippolito G, La Vecchia A, Umbrello G, Di Pietro G, Bono P, Scalia S (2021). Disappearance of seasonal respiratory viruses in children under two years old during COVID-19 pandemic: a monocentric retrospective study in Milan, Italy. Front Pediatrics.

[38] van Belkum A (2016). Hidden staphylococcus aureus carriage: overrated or underappreciated?. mBio.

[39] Mork RL, Hogan PG, Muenks CE, Boyle MG, Thompson RM, Sullivan ML (2020). Longitudinal, strain-specific Staphylococcus aureus introduction and transmission events in households of children with community-associated meticillin-resistant S aureus skin and soft tissue infection: a prospective cohort study. Lancet Infect Dis.

[40] Manara S, Pasolli E, Dolce D, Ravenni N, Campana S, Armanini F (2018). Whole-genome epidemiology, characterisation, and phylogenetic reconstruction of staphylococcus aureus strains in a paediatric hospital. Genome Med.

