# Correction: Epidemiology and antimicrobial susceptibility of Staphylococcus aureus in children in a tertiary care pediatric hospital in Milan, Italy, 2017—2021

**DOI:** 10.1186/s13052-022-01333-3

**Published:** 2022-07-29

**Authors:** Adriano La Vecchia, Giulio Ippolito, Vittoria Taccani, Elisabetta Gatti, Patrizia Bono, Silvia Bettocchi, Raffaella Pinzani, Claudia Tagliabue, Samantha Bosis, Paola Marchisio, Carlo Agostoni

**Affiliations:** 1 University of Milan, Milan, Italy; 2 Laboratory of Microbiology, Fondazione IRCCS Ca’ Granda Ospedale Maggiore Policlinico, Milan, Italy; 3 Pediatric Emergency Department, Fondazione IRCCS Ca’ Granda Ospedale Maggiore Policlinico, Milan, Italy; 4 De Marchi Foundation, Pediatric Area, Fondazione IRCCS Ca’ Granda Ospedale Maggiore Policlinico, Milan, Italy; 5 Fondazione IRCCS Ca’ Granda Ospedale Maggiore Policlinico, Pediatric Highly Intensive Care Unit, Milan, Italy; 6 Department of Pathophysiology and Transplantation, University of Milan, Milan, Italy; 7 Fondazione IRCCS Ca’ Granda Ospedale Maggiore Policlinico, Pediatric Intermediate Care Unit, via Francesco Sforza 9, 20122 Milan, Italy; 8 Department of Clinical Sciences and Community Health (DISCCO), University of Milan, Milan, Italy

**Correction: Ital J Pediatr 48, 67 (2022)**

**https://doi.org/10.1186/s13052-022-01262-1**

The original article [1] mistakenly omitted a Funding source which is noted in the statement ahead:

This study was partially funded by Italian Ministry of Health - Current research IRCCS.
